# Takotsubo cardiomyopathy secondary to intracranial hemorrhage

**DOI:** 10.1186/s12245-014-0033-4

**Published:** 2014-09-04

**Authors:** Mai Shimada, Jonathan D Rose

**Affiliations:** 1 Maimonides Medical Center, New York 11219, NY, USA; 2 Brookdale University Hospital and Medical Center, New York 11212, NY, USA

**Keywords:** Takotsubo, Cardiomyopathy, Apical ballooning syndrome, Subarachnoid hemorrhage, Intracranial hemorrhage, ECG abnormalities

## Abstract

Patients suffering from aneurysmal subarachnoid hemorrhage often present with electrocardiogram (ECG) abnormalities that mimic cardiac ischemia, but documented left ventricular regional wall-motion dysfunction has rarely been reported. This report is intended to raise the awareness of possible ECG changes secondary to subarachnoid hemorrhage (SAH). We cared for a 55-year-old female with an acute aneurysmal subarachnoid hemorrhage, whose evaluation was delayed and complicated by the presence of Takotsubo cardiomyopathy (TCM). Aneurysmal subarachnoid hemorrhage may induce Takotsubo cardiomyopathy that can present as an acute ST-elevation myocardial infarction. Physicians need to be aware of this possibility since it can lead to significant delays and treatment options for the patient.

## Background

Aneurysmal subarachnoid hemorrhage (SAH) has a reported mortality of 30 to 50%. Death results from the direct neurologic complications from the bleeding and cerebral ischemia. Although the mechanism has not been fully elucidated and may be due to neurohormonal activation and catecholamine surges, cardiac manifestations are commonly reported in the setting of SAH [1]. These may include electrocardiogram (ECG) changes of QT prolongation and T wave inversion, elevated biochemical markers of myocardial damage and heart failure, and decreased left ventricular function [1]-[3].

Takotsubo cardiomyopathy (TCM) is characterized by transient hypokinesis of the left ventricle that is often brought on by stress. It has been estimated that the incidence of TCM in SAH patients may be as high as 4 to 15% [4]. Since the treatment of TCM differs from that of acute myocardial infarction, it is important to be able to make the diagnosis early in the course of these patients. We report a case of a patient with aneurysmal subarachnoid hemorrhage whose evaluation and diagnosis were complicated by the presence of TCM. This case report illustrates the importance of recognizing TCM as a possible consequence of SAH.

## Case presentation

A 55-year-old female was at home sitting at a table when her family noted that she suddenly lost consciousness. Paramedics were called and found her unresponsive with agonal respirations. Her blood glucose was measured at 109 mg/dL, intubation was attempted but was not successful, and rescue breathing by bag-valve-mask was performed as she was transported to the hospital.

On arrival in the emergency department, the patient was unresponsive and making sonorous breath sounds. Her heart rate was 138 beats per minute, respirations 22 breaths per minute, blood pressure 154/103 mmHg, and oxygen saturation 96% on 100% O_2_. Unable to maintain her airway as a consequence of her mental status and believed to be in need of emergent CT imaging of her brain upon arrival, she was intubated. No evidence of head or body trauma was noted, and pupils were 4 mm bilaterally and equal but not reactive. Her Glasgow Coma Score was 9 (E4, V1, M4). The rest of her physical exam was unremarkable except for a healing incision over the left great toe.

Past medical history obtained from her family was significant for hypertension, and her current medications included enalapril, amlodipine, and metoprolol. They also noted that she had undergone a left foot surgery 3 weeks prior to her presentation. They denied any history of tobacco, alcohol, or illicit drug use.

**Figure 1:**
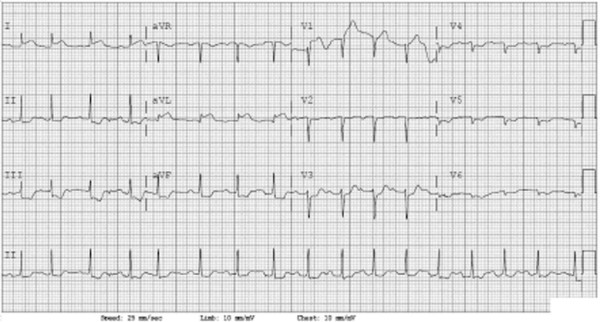
ECG obtained upon patient's arrival in the emergency department.

The patient was rapidly intubated and a stroke code called. At the same time, an ECG was obtained and revealed evidence of an inferolateral ST-segment elevation myocardial infarction (STEMI) (Figure 1). Although resulting after disposition, her initial laboratories revealed a pH of 7.26 and a lactate of 7.1 mmol/L. Serum chemistries demonstrated a sodium of 137 mmol/L, potassium of 3.4 mEq/L, chloride of 99 mEq/L, bicarbonate of 25 mEq/L, glucose of 234 mg/dL, creatinine of 0.9 mg/dL, and calcium of 9.2 mg/dL. The patient's white blood cell count was 15.6 × 103/uL, hemoglobin 14.2 g/dL, platelet count 298 × 103/uL, prothrombin time 10.6 s (INR of 0.9), and partial thromboplastin time 24.3 s. Her cardiac enzymes revealed a CKMB fraction of 3.4 units/L and a troponin I of 0.16 ng/mL (ref range 0.00 to 0.04).

Unstable and now with a suspected acute ST-elevation myocardial infarction, Cardiology was consulted and she was taken directly to the cardiac catheterization laboratory for revascularization prior to obtaining a head computer tomography (CT) scan. During the procedure, the patient received 2,500 units of heparin intraarterially but was not treated with any additional antiplatelet or anticoagulant therapy. The cardiac catheterization demonstrated normal coronary arteries with hypokinesis of the left ventricle.

**Figure 2:**
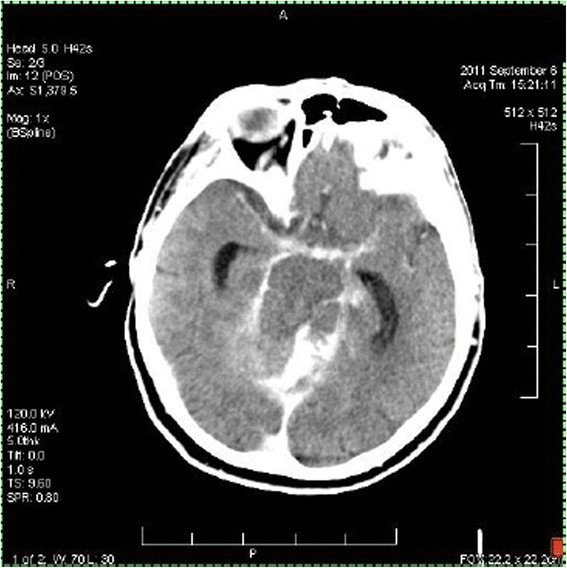
Noncontrast head computed tomogram obtained once the patient arrived in the intensive care unit.

Then, approximately 3 h after her arrival at the hospital, a noncontrast head CT was obtained that demonstrated a subarachnoid hemorrhage in the cisternal spaces around the cerebellum, in the prepontine and medullary cisterns and perimesencephalic and interpeduncular cisterns. This was associated with bilateral acute subdural hematomas, intraventricular hemorrhage, tonsillar and supratentorial herniation, and obstructive hydrocephalus (Figure 2). The patient was then transferred to the cardiac intensive care unit where Neurosurgery and Neurology were consulted and determined that she was clinically brain dead. With family involvement, assisted ventilation and vasopressor support was withdrawn and the patient expired on her fourth hospital day.

## Discussion

Aneurysmal subarachnoid hemorrhage carries a high mortality rate, and the main cause of morbidity and death is directly related to cerebral ischemia and intracranial mass effect [1]. ECG abnormalities have long been associated with the presentation of SAH, and elevations in troponin, CK-MB, and BNP are well-known harbingers of poor outcome [3],[5],[6]. It has been reported that 61.9% of patients with SAH will present with an abnormal ECG and that 20 to 30% of patients will manifest a secondary cardiomyopathy [7].

Several mechanisms for the cardiac complications that occur after SAH have been suggested. A generally accepted hypothesis is that the sympathetic stimulation induces catecholamine release in the myocardium leading to impaired systolic and diastolic function, repolarization abnormalities, and direct myocardial damage [3],[7],[8]. In addition, it has been reported that during the acute phase of SAH, there is an increase in aortic wall stiffness leading to higher left ventricular pressures [8]. These mechanisms may be responsible for the etiology for the TCM that occurred in our patient.

TCM most commonly occurs in women (89%) with a mean age of 68. Forty-six percent of cases are triggered by intense emotional or physical stress. The diagnostic criteria for TCM include the following: transient hypokinesis, akinesis, or dyskinesis in the left ventricular mid-wall segments with or without apical involvement; absence of obstructive coronary disease; new ECG abnormalities or modest elevation in cardiac troponin; and absence of pheochromocytoma and myocarditis [9]. In general, TCM is a transient disorder that can be managed with supportive therapy such as angiotensin-converting-enzyme (ACE) inhibitors, beta-blockers, and diuretics [4],[10]. Aspirin has also been suggested in the presence of coexisting coronary atherosclerosis [10],[11].

Our case represented a delay to diagnosis in a patient with TCM who appeared to be suffering from an acute ST-elevation myocardial infarction. Although the initial differential diagnosis included the potential for a cerebral vascular event, the presence of STEMI criteria on her ECG along with her unstable condition led us to investigate her apparent cardiac pathology in the catheterization lab prior to obtaining a head CT. Given the patient's poor prognosis when she arrived, it is unclear if the heparin she received during cardiac catheterization worsened her SAH or changed her outcome. As emergency physicians, we need to be aware of the danger of anchoring to a single diagnosis, which can easily blind us from other possible pathologies.

## Conclusions

Aneurysmal subarachnoid hemorrhage may induce Takotsubo cardiomyopathy that can present as an acute ST-elevation myocardial infarction. Physicians need to be aware of this possibility since it can lead to significant delays and treatment options for the patient.

## Competing interests

The authors declare that they have no competing interests.

## Authors’ contributions

JR cared for the patient and participated in editing the manuscript. MS conceived of and drafted the manuscript and is responsible for its contents. Both authors read and approved the final manuscript.
